# Tar Spot on Maize: Impact of Soil Types and Environmental Conditions on the Survival of *Phyllachora maydis* in the Subtropical Climate of Florida

**DOI:** 10.3390/jof11060443

**Published:** 2025-06-11

**Authors:** Vitor A. S. Moura, Larissa C. Ferreira, Marcio F. R. Resende, Katia V. Xavier

**Affiliations:** 1 Everglades Research and Education Center, University of Florida/IFAS, Belle Glade, FL 33430, USA; v.silvademoura@ufl.edu (V.A.S.M.); larissacarvalhof@ufl.edu (L.C.F.); 2 Horticultural Sciences Department, University of Florida/IFAS, Gainesville, FL 32611, USA; mresende@ufl.edu

**Keywords:** South Florida, corn, sweet corn, histosol, krome, ascospore viability, spore germination, tillage, Palm Beach County, Miami-Dade County

## Abstract

Tar spot, caused by *Phyllachora maydis*, is an established maize disease in the Midwest of the United States but remains an emerging concern in Florida. While this pathogen can overwinter on plant residue, its survival in Florida’s subtropical environment is not well understood. This study evaluated how environmental factors affect the germination of *P. maydis* ascospores and stroma integrity. Symptomatic maize leaves were incubated under four conditions: Histosol soil (muck), Krome soil (rocky), 4 °C, and 23 °C. Extensive leaf decomposition occurred in both soil types, with most plant material degraded after eight weeks, while the stroma maintained its structure. Despite this, ascospore germination declined across all conditions. After eight weeks, ascospores incubated at 4 °C retained 25% viability, while those at 23 °C had the lowest germination (0.7%). Ascospores from leaves buried in soil exhibited low viability (1–6%), with no significant differences between soil types (p=0.9944). Weather analysis revealed that increased temperature reduced germination rates, while higher humidity enhanced them. These findings suggest that *P. maydis* displays limited survivability under Florida-like conditions, with germination rates declining over time. Therefore, cultural practices such as tillage, already employed by corn producers in Florida, may be effective in reducing sources of *P. maydis* inoculum.

## 1. Introduction

Maize (*Zea mays* L.) is characterized by its various uses, easiness to cultivate and store, large yield potential, and high starch content in the grain [1,2]. In Florida, maize production consists of sweet corn, valued for its fresh consumption, and field corn, primarily used for livestock feed and industrial purposes [1,2,3]. In 2024, Florida was the largest producer of fresh sweet corn in the USA and contributed approximately $293 million to the value of production [4]. While not a major field corn producer in the USA, the state still generated around $30 million from field corn [4].

Tar spot, caused by *Phyllachora maydis*, is a significant foliar disease impacting maize production in the Midwest of the USA. Since its initial detection in Illinois and Indiana in 2015, tar spot has rapidly spread, leading to substantial yield losses [5,6]. Between 2018 and 2020, USA losses were estimated at 241 million bushels [5]. During the 2018 growing season, an epidemic of tar spot in the Midwest caused 25 to 30% yield losses [5,7,8]. By 2021, tar spot had become the most destructive corn disease in the USA, resulting in a loss of 231 million bushels [9]. It remained the most significant corn disease in both 2022 and 2024, although it ranked sixth in 2023 [10,11,12]. The disease has also emerged as a growing concern in Florida since its first report in 2016 [13,14].

The symptoms and signs include the formation of characteristic black, tar-like structures on infected corn leaves, named stroma [15,16]. In temperate regions, such as the Midwest of the USA, the pathogen is known to overwinter on plant residue, with ascospores serving as the primary inoculum source for subsequent growing seasons [17,18]. Groves et al. [17] assessed ascospore viability in overwintered tar spot-infected material collected from fields with both till and no-till management regimes across various states in the Midwest, finding viability ranging from 2.5 to 25%. However, the survival and viability of *P. maydis* under the warmer and more humid conditions of the subtropical climate of Florida remain unexplored. Temperature is a key factor influencing pathogen persistence, with cooler conditions generally favoring the survival of ascospores, while prolonged exposure to high temperatures and fluctuating humidity can reduce their viability. This trend is supported by multiple studies showing that fungal spore survival is strongly influenced by temperature. For example, *Beauveria bassiana* conidia remained viable for up to 276 days at 10 °C, while *Fusarium solani* chlamydospores retained over 50% germinability for 70 days under similar low-temperature conditions [19,20]. In contrast, higher temperatures (≥25–30 °C) consistently led to rapid declines in viability, with spores of *B. bassiana* and *Colletotrichum acutatum* losing viability rapidly at 25 °C and becaming unrecoverable at 55 °C [19,21]. In addition to temperature, soil properties and management practices strongly influence the decomposition of leaf residues and the survival of fungal pathogens, especially when infected tissues are incorporated into the soil through practices such as tillage [22]. Unlike the Midwest, where no-till or minimum tillage systems are widely adopted [23], South Florida relies primarily on conventional tillage. The region is also characterized by diverse soil types and the summer season serving as the corn off-season [24,25,26]. Miami-Dade and Palm Beach Counties are the major sweet corn-producing areas in the state, with a total cultivation area of 28,000 ha [27,28]. These areas are characterized by distinct soil types: Histosol soil, commonly known as muck soil, in Palm Beach County and Krome soil, very gravelly loam, often referred to as rocky soils, in Miami-Dade County [25,26]. Muck soils, or histosols, are organic-rich and can have an organic matter content greater than 80%, while Krome soils, a type of calcareous rocky soil, are rock-plowed soils that have gravelly textures with organic matter usually less than 2% [25,26]. Soil texture can influence the rate of leaf decomposition and, consequently, the survival of foliar pathogens. For instance, fine-textured, organic-rich soils like muck tend to promote faster decomposition of infected leaf tissue, partly due to higher microbial activity and enzyme production, such as acid phosphatase, which has been shown to correlate with increased fungal biomass and decomposition capacity in organic soils [29].

Although tar spot is economically significant in other parts of the country, it remains an emerging issue in Florida, where, as of 2024, it has had a limited impact on sweet corn production [13]. Given the economic importance of sweet corn in South Florida and the potential for tar spot to become a greater threat, it is crucial to investigate how various environmental conditions, especially soil types, influence the survivability of *P. maydis* in Florida. Previous research conducted in the Midwest focused on how freezing temperatures and minimal tillage affected *P. maydis* survival [17]. However, the considerable differences in climate and soil conditions between the Midwest and Florida prompt the need to evaluate *P. maydis* survivability under Florida conditions. In addition, this study aims to provide novel insights into the effects of temperature and time after leaf detachment on the viability of *Phyllachora maydis* ascospores. The objectives of this study were (1) to assess the effect of specific environmental factors, such as temperature, humidity, rainfall, seasonal variation, and natural soil biotic activity, on *P. maydis* survival and tar spot-related leaf decomposition; (2) to determine the effects of soil types and environmental conditions on *P. maydis* ascospore germination; and (3) to evaluate the temporal impact on ascospore germination post-leaf detachment.

## 2. Materials and Methods

### 2.1. Experimental Design

**Figure 1:**
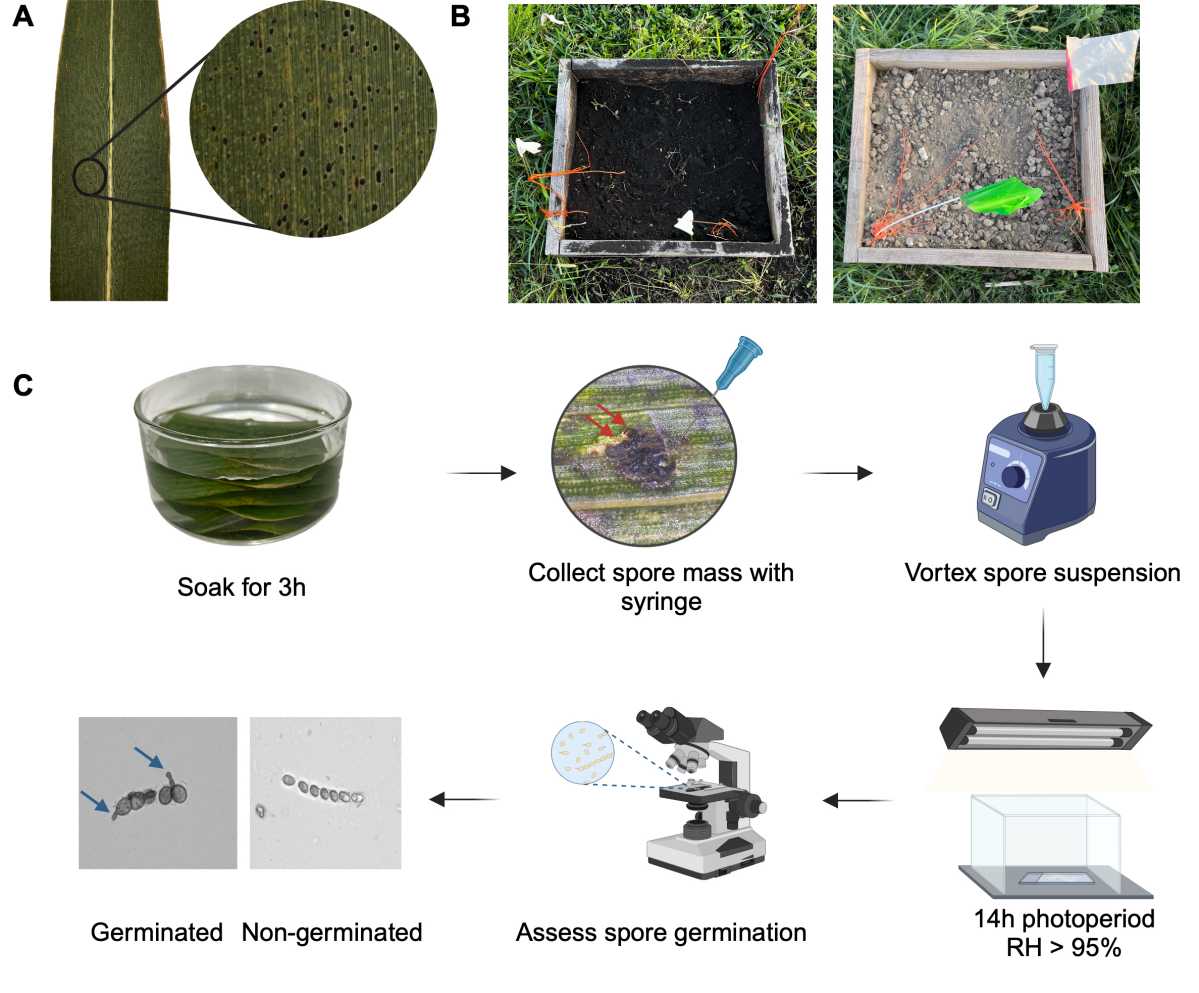
Workflow illustrating the experimental process. (**A**) Maize leaf displaying typical tar spot symptoms caused by *Phyllachora maydis* in Florida. (**B**) Wooden frames filled with muck soil (**left**) and rocky soil (**right**) used for leaf incubation. (**C**) Evaluation process of ascospore germination. After leaf retrieval from incubation, samples underwent washing and surface sterilization before being soaked in water for 3 h. Spore masses (indicated by red arrows) were collected using a syringe, and the suspension was vortexed to ensure homogenization. The suspension was then incubated in a humidity chamber on concave glass slides for 24 h under a 14 h photoperiod. Ascospore germination was assessed, with spores displaying a germ tube (indicated by blue arrows) classified as germinated. Created in BioRender.com.

The survival of *Phyllachora maydis* was investigated under four environmental conditions (treatments): muck soil, rocky soil, 4 °C, and 23 °C. To assess the survival of *P. maydis* across these treatments, symptomatic maize leaves displaying typical tar spot symptoms (Figure 1A) were collected from three fields in Miami-Dade County and brought to the laboratory for further processing. For the 4 °C treatment, leaves were stored in paper bags at 4 °C in a laboratory refrigerator for the duration of the experiment. For the 23 °C treatment, leaves were pressed and dried onto herbarium paper sheets and stored at room temperature, which fluctuated within a range of 23 °C ± 3 °C. For soil incubation treatments, 10 leaves were individually placed into 200-micron nylon mesh bags and buried approximately 20 cm deep, simulating tillage depth, in open-bottomed wooden frames (0.5 m × 0.6 m × 0.25 m) filled with either muck or rocky soil (Figure 1B). The muck soil was collected from the Everglades Research and Education Center (EREC), while the rocky soil was brought from Miami-Dade County. These frames were placed at EREC (26°39′25.2″ N, 80°37′48.8″ W) directly on the ground to allow interaction with the underlying soil while still containing the samples. The site is located adjacent to a Florida Automated Weather Network (FAWN) weather station.

The experiment was repeated twice in 2023 and once in 2024, each lasting approximately eight weeks: (i) mid-May to mid-July 2023, (ii) late July to late September 2023, and (iii) mid-February to mid-April 2024. A treatment was added in the third repetition, where leaves were stored in plastic bags at −20 °C.

### 2.2. Assessment of Leaf Decomposition, Tar Spot Stromata Integrity, and Ascospore Viability

#### 2.2.1. Leaf Retrieval and Sampling Procedures

Leaf samples were retrieved from incubation under the four treatment conditions to assess leaf decomposition, tar spot stroma integrity, and ascospore germination. Sampling was conducted biweekly, at 2, 4, 6, and 8 weeks, with the final time point corresponding to when the plant material was largely degraded. At weeks 2 and 4, one leaf was collected from each treatment and considered a single experimental unit. At weeks 6 and 8, due to advanced decomposition, three leaves were collected from each soil treatment and combined to form one experimental unit. For the indoor treatments, leaf segments from approximately three leaves were collected to form an experimental unit, ensuring a comparable amount of tissue to that retrieved from the soil treatments.

#### 2.2.2. Leaf Decomposition and Tar Spot Stroma Integrity Assessment

After collection, leaves were first visually assessed and photographed to evaluate overall decomposition. The assessment considered the presence of holes, degree of fragmentation, appearance, and color of remaining tissue, the extent of connecting fibers between leaf segments, the fragility of the tissue when handled, and the overall amount of remaining leaf material, providing a qualitative estimate of decomposition severity. Then, the samples were washed in soapy water and rinsed with running water to remove soap and soil debris. During washing, loose leaf segments were retained using a 16-mesh sieve (1.18 mm; Thermo Fisher Scientific, Waltham, MA, USA). Next, stroma integrity was examined under a Leica EZ4E stereo microscope (Leica Microsystems, Wetzlar, Germany) and photographed with its integrated camera. This analysis was primarily qualitative and focused on visible features such as overall appearance, presence of holes, or any distinctive deviations from the structure of an intact, regular stroma.

#### 2.2.3. Ascospore Germination Assay and Quantification

The washed leaves underwent surface sterilization by soaking the leaves in 70% ethanol for 30 s, followed by 0.6% sodium hypochlorite for 2 min, and then rinsed three times with autoclaved distilled water. The leaves were then soaked in autoclaved distilled water for three hours to stimulate ascospore exudation. The spore mass was harvested from both the stromatal exudate and the internal stromata compartments using a needle. For the soil treatments, the entire stroma was collected and crushed, as no exudates were produced, and the stroma easily disintegrated upon contact. The spores were then placed into a microcentrifuge tube containing 300 µL of autoclaved deionized water and resuspended by vortexing for 1 min to disrupt spore clusters. The ascospore concentration was then adjusted to a range from 5 × 10^4^ to 5 × 10^5^ ascospores/mL.

The spore suspension was subdivided into four replicates, with each replicate consisting of a 60 µL aliquot pipetted onto concave glass slides. The slides were then incubated for 24 h inside a covered humidity chamber (relative humidity > 95%, 23 °C ± 2 °C) under a 14 h photoperiod. Germination was assessed under 40× magnification using a Leica DM750 microscope (Leica Microsystems, Wetzlar, Germany) in a binary system, categorizing spores as either germinated or non-germinated. An ascospore was considered germinated if it had formed a germ tube extended to at least half the length of the spore (Figure 1C). To minimize bias from spore clustering, germination was evaluated in ten random fields of view per replicate, with at least 40 ascospores assessed per treatment. In the third experiment, ascospores were harvested immediately after leaf collection, processed in the same manner, and incubated for 24 h on concave slides, with germination rates used as the baseline reference.

### 2.3. Weather Data Processing

Weather data were obtained from the FAWN station in Belle Glade, FL, from March 2023 to April 2024. Observations collected every 15 min included air temperature (2 m height), soil temperature (10 cm depth), relative humidity, and rainfall. Soil temperature was measured using a 107-L probe (Campbell Scientific, Logan, UT, USA); air temperature and relative humidity were recorded with a CS215 sensor and HygroVUE 5/10 sensor (Campbell Scientific, Logan, UT, USA); and rainfall was measured using a TB3/TB4 tipping-bucket rain gauge (Hydrological Services, Sydney, NSW, Australia) and a 370D tipping-bucket rain gauge (Met One Instruments, Grants Pass, OR, USA). For each experiment and month, the average, minimum, and maximum values were calculated for temperature and humidity, while rainfall was summed.

### 2.4. Statistical Analysis

All analyses were performed in R v.4.2.2 using the packages glmmTMB, car, emmeans, and DHARMa [30,31,32,33]. Germination was expressed as the proportion of germinated ascospores out of the total counted and analyzed using a generalized linear mixed model (GLMM) with a beta-binomial distribution and logit link. Model residuals were validated using the DHARMa package.

Fixed effects included treatment, week, and their interaction. Type II Wald $\chi^{2}$ tests (from the car package) assessed the significance of fixed effects. To examine the effect of soil types and environmental conditions on *P. maydis* ascospore germination, estimated marginal means (EMMs) were computed using emmeans with a Treatment | Week specification and Tukey-adjusted *p*-values for post-hoc comparisons. To assess the temporal impact on ascospore germination after leaf detachment independent of treatment, EMMs were computed for the main effect of Week using the same model. Statistical comparisons of germination probabilities between the initial (week two) and final (week eight) time points within each treatment were performed using pairwise contrasts of EMMs derived from the same model. Specifically, contrasts were defined to compare week eight against week two for each treatment group individually.

Additionally, germination data from the baseline and −20 °C frozen treatments were appended to the main dataset and analyzed similarly using a GLMM with a beta-binomial distribution and logit link, with a combined treatment-week factor included as the fixed effect. Pairwise comparisons of estimated marginal means were conducted to test the effect of each treatment at eight weeks relative to the baseline.

To assess the impact of weather conditions on ascospore germination, a separate GLMM was fitted using germination proportion (from both muck and rocky soil treatments) as the response variable. Weather variables (soil temperature, relative humidity, and rainfall) were scaled and centered and included as fixed effects. These weather data corresponded to the two weeks preceding each sampling date. Type II Wald $\chi^{2}$ tests were used to assess the significance of fixed effects, and EMMs were calculated for post-hoc contrasts, as previously described.

## 3. Results

### 3.1. Seasonal Variations and Environmental Factors Affecting *Phyllachora maydis* Survival and Tar Spot-Related Leaf Decomposition

Leaves with tar spot were visually inspected upon retrieval from incubation under each condition. Leaves incubated in soil showed clear signs of degradation, while those kept indoors at 4 °C and 23 °C remained largely intact throughout the 8-week experiment. When focusing on the leaves incubated in soil, decomposition of maize leaves with tar spot varied significantly across seasons, with notable differences between the spring/summer and winter/spring incubation periods.

**Figure 2:**
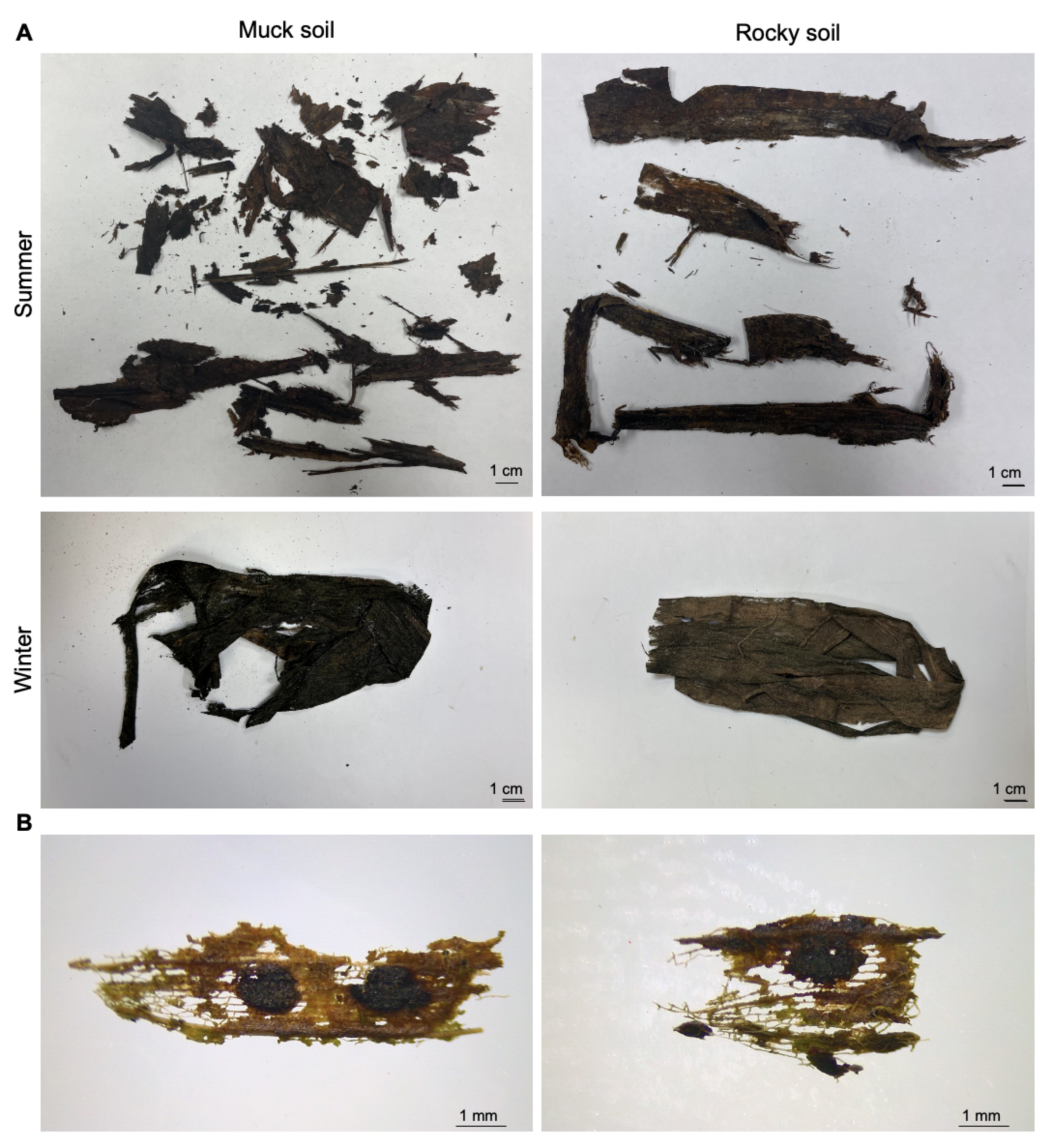
Images of tar spot-infected leaves after incubation in different soils in Florida. (**A**) Leaf incubated in muck soil (**left**) during summer and in rocky soil (**right**) during winter for four weeks. Scale bars represent 1 cm. (**B**) Representative images of stroma at various time points up to 1 month of incubation. Scale bars represent 1 mm.

During the spring/summer 2023 period, significant degradation was observed after four weeks of incubation, with leaves breaking down considerably and many fragments detaching from the main structure. As the softer leaf tissue decomposed, the veins became more exposed, leaving behind a skeletal framework (Figure 2A). By the end of the eight-week incubation period, the leaves were mostly decayed. In contrast, during the winter/spring experiment, the leaves showed significantly less degradation after four weeks of incubation. These leaves retained much of their original structure, with minor signs of decomposition (Figure 2A).

We observed that leaves incubated in the muck soil were more degraded than those in rocky soil, regardless of the season (Figure 2A). Interestingly, recovered tar spot stromata retained their shape with only some occasional holes in both soil types, showing less degradation compared with the surrounding leaf tissue (Figure 2B). The overall integrity of stromata did not differ visually between the different time points and seasons.

The leaves incubated at 4 °C and 23 °C were maintained under controlled conditions with minimal fluctuations in temperature and humidity. In contrast, the leaves incubated in soil were exposed to variations in temperature, humidity, and rainfall, as well as natural factors in the soil, such as the microbiome and insects.

**Figure 3:**
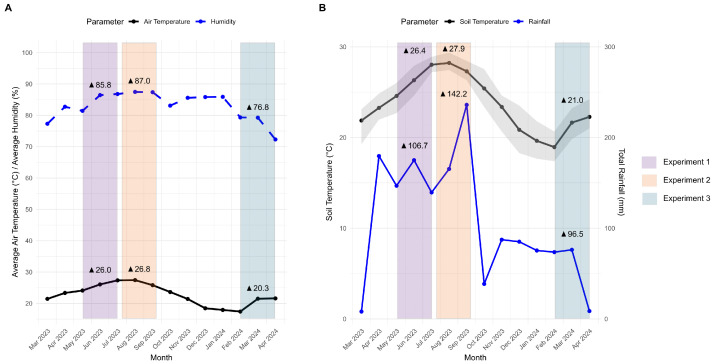
Environmental conditions recorded across different experiments from March 2023 to April 2024. (**A**) Monthly average air temperature (°C) and relative humidity (%). (**B**) Monthly average soil temperature (°C) and total rainfall (mm). The shaded area represents the maximum and minimum soil temperatures recorded each month. Colored bars indicate weather data corresponding to experiments 1, 2, and 3. Triangles denote the average values for each parameter during each experiment.

To better understand the seasonal effects on samples incubated in muck and rocky soils, we analyzed the weather data collected at the experiment site. During experiment 1 (spring/summer 2023), the weather conditions were characterized by an average air temperature of 26.0 °C and high humidity (85.8%, Figure 3A), with a soil temperature of 26.4 °C and total rainfall of 106.7 mm (Figure 3B). Experiment 2 (summer 2023) was warmer, with an average air temperature of 26.8 °C, humidity of 87.0%, and total rainfall of 142.2 mm. The soil temperature rose to 27.9 °C. In contrast, experiment 3 (winter/spring 2024) had an average air temperature of 20.3 °C, humidity of 76.8%, soil temperature of 21.0 °C, and total rainfall of 96.5 mm.

### 3.2. Effect of Soil Types and Environmental Conditions on *P. maydis* Ascospore Germination

**Figure 4:**
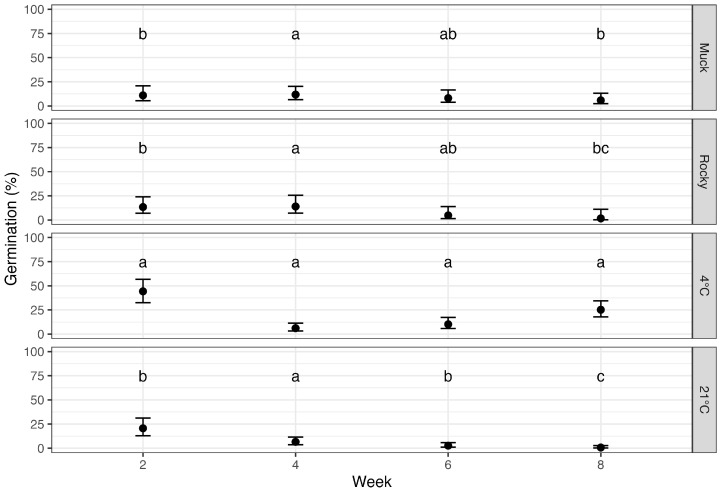
Germination rates of *Phyllachora maydis* ascospores across different soil types and environmental conditions (muck soil, rocky soil, 4 °C and 23 °C) over incubation weeks. The x-axis represents the number of weeks of incubation, and the y-axis shows the probability of germination. Error bars represent 95% confidence intervals. Data collected at each time point were pooled across seasons for analysis. Letters above the bars indicate statistical significance from pairwise comparisons conducted within each time point; treatments sharing the same letter are not significantly different at that specific time point.

Ascospore germination was measured biweekly to assess the effect of soil types and environmental conditions over time, with “time” indicated by the number of weeks a sample was incubated after leaf detachment. Data collected at each time point were pooled across seasons. Treatments, time of incubation, and their interaction significantly affected ascospore germination (*p* < 0.0001), with a general trend of decreasing germination over time for all conditions (Figure 4).

At two weeks post-incubation, the samples incubated at 23 °C, rocky and muck soils exhibited low germination rates (20.6%, 13.4%, and 10.9%, respectively), which were not significantly different. Samples incubated at 4 °C had significantly higher germination rates of 44.2% (p<0.05). By eight weeks of incubation, germination rates had decreased to 1–6% in both soil types as well as incubation at 23 °C, while incubation at 4 °C retained 25.2% germination.

**Figure 5:**
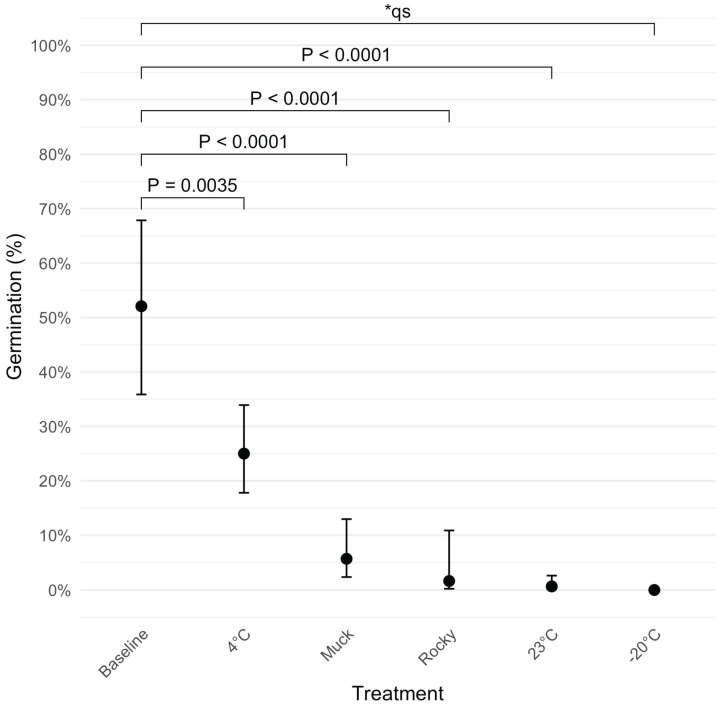
Estimated germination probabilities for various treatments (muck soil, rocky soil, 4 °C, 23 °C, and −20 °C) after eight weeks of incubation, compared with the baseline. A baseline germination rate was established using fresh samples collected on the day of field sampling. Each point represents the estimated germination probability for the respective treatment, with error bars indicating 95% confidence intervals. *p*-values for pairwise comparisons between the 8-week treatments and the baseline are shown above the points. * qs indicates quasi-complete separation.

To evaluate the impact of incubation on ascospore viability, we compared the results from each treatment to a baseline germination rate, which was obtained from ascospores collected from freshly harvested infected leaves without any incubation treatment (Figure 5). After eight weeks of incubation, germination dropped from 52.1% to 5.7% and 1.7% in the muck and rocky soil treatments, respectively (p<0.0001), representing decreases of 89.1% and 96.8%. The 23 °C incubation treatment had the greatest reduction (to 0.7%, a 98.7% loss; p<0.0001). Incubation at 4 °C had the smallest decline (52.0%; p=0.0035). No germination (0%) was observed in the −20 °C treatment.

Germination rates did not differ significantly between muck and rocky soil treatments (p=0.9944), so data from both were combined to evaluate the influence of weather variables. Due to the high correlation between air and soil temperatures (r=0.98), air temperature was excluded from the final model to avoid multicollinearity. The model indicated that soil temperature had a significant negative effect on germination (β=−1.0556, p=0.0177), while rainfall had a marginally negative effect (β=−0.3013, p=0.0736). In contrast, humidity showed a significant positive effect (β=2.0566, p=0.0046).

### 3.3. Temporal Impact on Ascospore Germination Post-Leaf Detachment

**Figure 6:**
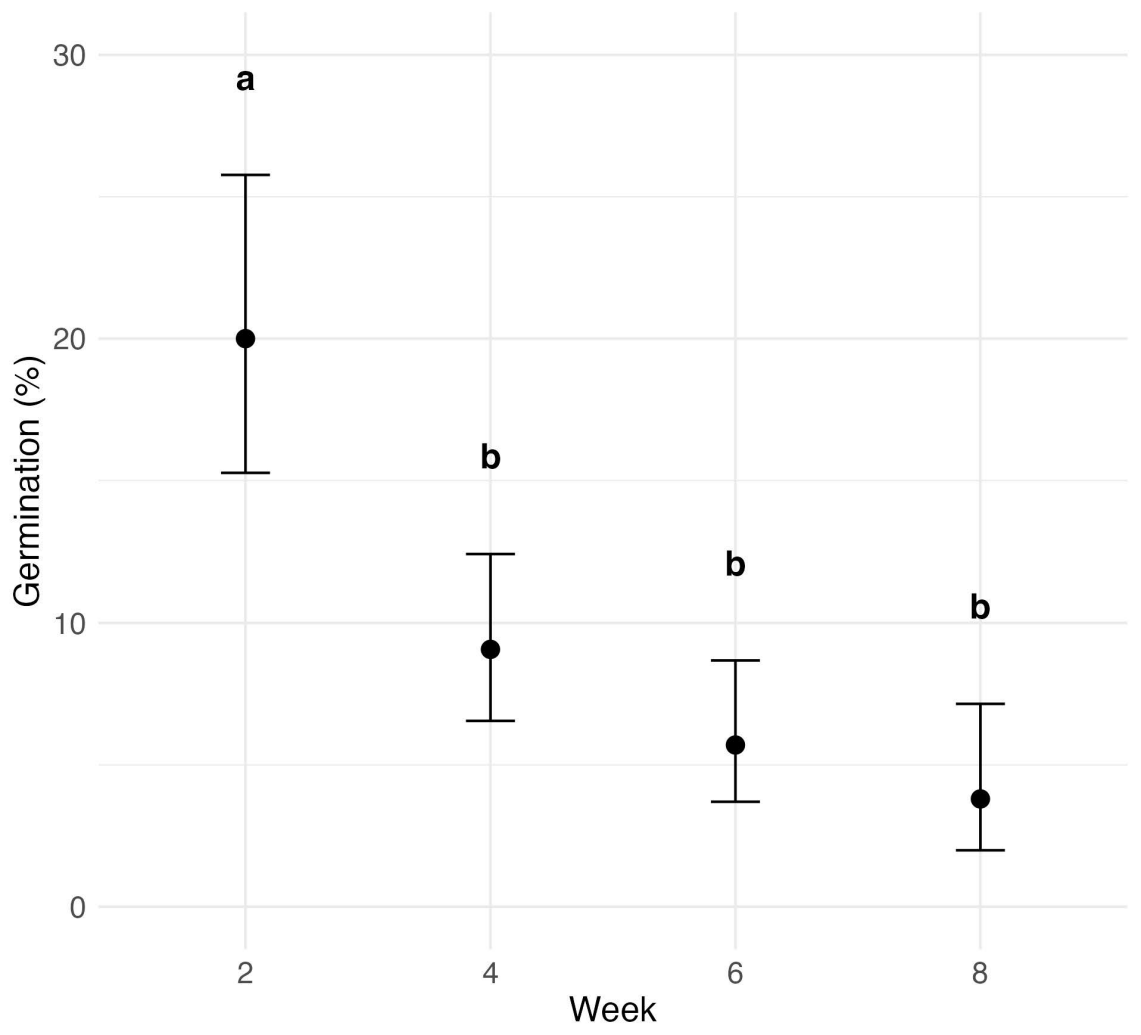
Effect of time after leaf detachment on *P. maydis* ascospore germination across all treatments (muck soil, rocky soil, 4 °C, and 23 °C). The x-axis represents the number of weeks that samples were incubated after leaf detachment, and the y-axis shows the germination probability, with error bars indicating 95% confidence intervals. Letters above the bars indicate statistical significance from pairwise comparisons; treatments with the same letter are not significantly different.

The effect of time on ascospore germination was assessed across all treatments (Figure 6). Germination consistently declined over time, with the most significant drop by week four (p<0.0001). Germination at week two was significantly higher than at all subsequent time points. Although germination continued to decrease beyond week four, the differences between weeks four, six, and eight were not statistically significant, indicating a progressively slower rate of decline.

A comparison of final germination rates at eight weeks with the initial rates at week two revealed further insights (Table 1). At 4 °C, germination retained 42.4% of its initial value (57.6% decline; p=0.0113). In contrast, 23 °C resulted in only 2.7% of the original rate remaining, a 97.3% decline (p<0.0001). Rocky soil showed an 88.9% reduction, while muck soil had a moderate 50.1% decrease, which was not statistically significant (p=0.2501). Initial germination in the muck soil was already low (10.9%), and final germination remained similarly low.

## 4. Discussion

This study investigated the survival and germination dynamics of *Phyllachora maydis* under varying environmental conditions and timeframes. We found that seasonal variation significantly influenced leaf decomposition and tar spot stroma integrity, with faster degradation occurring during the summer. Soil conditions also affected ascospore germination, promoting lower germination rates in comparison to leaves kept at 4 °C. Furthermore, germination declined over time after leaf detachment, suggesting a limited window of ascospore viability.

The seasonal effect on leaf degradation was evident across experimental periods, with our results showing markedly faster decomposition during the summer compared with the winter/spring. This finding is consistent with previous studies by Huang et al. [34,35], which reported that maize residue decomposition is accelerated under warmer temperatures due to heightened microbial activity. Higher temperatures stimulate microbial metabolism and growth rates, thereby increasing the abundance and activity of decomposer organisms such as bacteria and fungi [36]. These microbes break down complex organic compounds in plant residues, including cellulose and lignin, leading to more rapid decomposition [37]. Additionally, warmer conditions tend to increase enzymatic activity and nutrient cycling, further facilitating microbial degradation processes [38,39,40]. In our summer 2023 experiment, leaves in both muck and rocky soil treatments were nearly fully decomposed after eight weeks, whereas in the winter/spring 2024 experiment, plant material remained significantly more intact. This difference likely reflects the influence of environmental conditions, particularly temperature, on microbial-mediated degradation processes. These results reinforce the importance of seasonal variability in shaping residue persistence and, consequently, the survival dynamics of *P. maydis* within decomposing tar spot stromata. However, this study was conducted under field-simulated conditions in soil incubation containers, which may not fully capture all field-scale variables; future studies should explore in situ decomposition across seasons and geographic regions to validate these findings.

The soil types evaluated in this study, muck and rocky soil, represent two common soil types in the Florida agricultural landscape, particularly in the two major corn-producing counties in South Florida, where tillage is widely practiced [26,41]. Leaves incubated in muck soil exhibited significantly greater degradation compared with those in rocky soil at the same time point, but the stromata retrieved from both soils were in similar conditions. We observed some occasional holes in the stromata, which were very fragile and would easily disintegrate upon contact. Despite differences in leaf degradation, stroma integrity did not influence ascospore viability. Our results indicate that *P. maydis* ascospores exhibit limited survival in both muck and rocky soils, with germination rates declining over time, and no significant difference was found between the two soil types in the rate of germination decline. These findings are consistent with previous research showing that soil microbial activity and environmental conditions can significantly influence pathogen survival. Muck soils are rich in organic matter (∼80%), which supports microbial activity that can facilitate the breakdown of plant residues and reduce the inoculum potential [26,41]. Tillage practices further enhance microbial oxidation and decomposition [41,42]. This is supported by our observation that germination rates in muck soil dropped sharply within the first two weeks, suggesting rapid microbial-mediated degradation. In contrast, rocky soils, which are derived from limestone parent material and characterized by low organic matter (<2%), alkaline pH, and limited water retention capacity, are less conducive to microbial activity [25,26,43]. Lohila et al. [42] reported that soil respiration (CO_2_) in histosols was three times higher than in sandy soil with lower organic matter content (<2%), reinforcing the role of microbial activity in organic soils. Despite differences in microbial activity and decomposition potential between the two soil types, these did not translate into significant differences in ascospore germination. In rocky soil, the lack of soil moisture and the potential for desiccation may accelerate pathogen degradation and limit its ability to survive [44]. In this context, the survival of *P. maydis* in rocky soils is likely influenced by moisture retention, with the pathogen’s ability to survive on plant residues being further challenged in drier conditions. This observation is further supported by the notably low germination rate under 23 °C, a condition under which the leaves were dried for herbarium preservation. Additionally, while humidity has a significant positive effect on spore germination, excessive rainfall can have a marginally negative impact on germination. In rocky soils, where water retention is already minimal, the presence of excessive rainfall could exacerbate drying and leaching effects, further hindering the survival ability of *P. maydis* and the ability of its ascospores to germinate. However, the moisture from rainfall may also trigger the exudation of *P. maydis* ascospores [45], facilitating ascospore germination in the soil. By the time of assessment, those ascospores with potential germination viability may have already been discharged, limiting the observed germination at later stages. This is also evidenced by the increasing difficulty in detecting ascospores in later assessments in soil. Therefore, the viability of *P. maydis* in these soils likely depends on maintaining a balance of moisture levels that support its survival while avoiding conditions that lead to desiccation or waterlogging.

Temperature and moisture conditions had a critical influence on *P. maydis* ascospore viability in this study. Leaves incubated at 4 °C mantained significantly higher germination rates than all other conditions, with viability remaining around 25.2% after eight weeks. In contrast, incubation at 23 °C, where leaves were rapidly dried by pressing, resulted in a dramatic decline in germination, with only 2.7% of the initial rate remaining by the end of the study. This highlights the negative impact of warmer, dry conditions on spore viability, consistent with findings by Solórzano et al. [15]. In the 4 °C condition, moisture was retained longer due to gradual drying, which may have helped preserve viability. Conversely, the −20 °C treatment led to a complete loss of germination after eight weeks, a finding that contrasts with earlier studies reporting viable spores after similar freezing conditions [15,17,46]. This discrepancy may arise from differences in experimental conditions or natural variations in stroma/spore maturity. Previous research by Parbery [47] demonstrated that *P. maydis* perithecia contain asci at various stages of development and can discharge ascospores repeatedly under favorable conditions. Consequently, the maturity of stromata at the time of sampling likely influenced the availability and viability of ascospores throughout the experiment. Because our samples included stromata at different developmental stages, variability in ascospore maturity may have contributed to irregular germination patterns over time. However, there is currently a lack of data on how storage or environmental conditions affect stromatal maturation, and this represents a key gap in our understanding. Future studies should aim to characterize stromatal maturity at sampling and explore how environmental factors influence the development and viability of *P. maydis* ascospores.

The observed decline in *Phyllachora maydis* ascospore germination over time supports previous findings that this pathogen can survive on crop residue, but it extends this knowledge by quantifying the steep and time-sensitive loss of spore viability under subtropical conditions. In contrast to studies from temperate regions, where ascospores have been shown to remain viable through prolonged cold winters, with viability ranging from 2.5% to 25% at temperatures as low as −34 °C [17,48]. Our results demonstrate a much faster decline in viability under warm storage. This temporal loss was especially pronounced between weeks two and four post-detachment, after which germination plateaued at low levels regardless of treatment, indicating a critical early window for viability loss. Cooler storage at 4 °C slowed this decline, preserving 42.4% of the initial germination potential by week eight, while at 23 °C, viability dropped to just 2.7% of the starting value. This temporal perspective adds crucial context for understanding regional differences in disease pressure and informs management strategies that minimize the early-season risk of tar spot outbreaks. However, since these data were generated under controlled conditions, future field studies are needed to validate how microbial interactions, rainfall, and sunlight further influence the time-dependent decline of *P. maydis* viability on crop debris.

While this study provides important insights into the survival and germination of tar spot stromata under controlled incubation conditions, some limitations should be acknowledged when applying these findings to field scenarios. The use of open-bottomed wooden frames filled with muck or rocky soils helped simulate certain aspects of natural environments but may not fully capture the complexity of in situ soil conditions, including microbial activity. Additionally, the partially above-ground incubation may have influenced temperature and moisture dynamics differently than fully buried conditions of tilled soils. Nonetheless, the observed survival of stromata for up to two months suggests limited potential for persistence in South Florida.

Our findings provide a valuable understanding of the potential survival and epidemiology of *P. maydis* in the Florida subtropical environment. Unlike the Midwestern region of the USA, where tar spot overwinters and spreads with more prolonged ascospore viability, the warm, humid climate of South Florida may not support long-term ascospore viability. Tillage is a widely adopted practice in South Florida and is also considered a cultural strategy to reduce tar spot inoculum [49]. However, its effectiveness in minimizing initial inoculum levels may be constrained by inconsistent adoption at the local scale, as observed in a recent study conducted in the Midwest [50]. For example, a corn producer who uses tillage to incorporate infected maize residue into the soil may still experience disease pressure if neighboring fields remain under no-tillage management, as this can allow airborne spores to persist and spread. Considering that tar spot is still an emerging disease in Florida with limited impact on sweet corn production as of 2024 [13,51], its late-season onset suggests that the widespread use of tillage, coupled with the warm off-season climate in the state, may play a role in mitigating its establishment and spread. However, further research is needed to fully understand how these practices, in combination with the subtropical conditions in Florida, influence tar spot disease dynamics in the field. Understanding the interactions between regional tillage practices, climate conditions, and disease epidemiology will be crucial for developing effective, long-term management strategies for tar spot.

## 5. Conclusions

This study provides new insights into the survivability of *Phyllachora maydis* under different environmental conditions following leaf detachment, including incubation at 4 °C, 23 °C, and muck and rocky soils. Ascospores recovered from leaves incubated in rocky and muck soils experienced substantial viability losses (96.8% and 89.1% declines, respectively), though stromata structures remained visually intact across all treatments throughout the two-month experiment. By the end of this period, leaves incubated in soil were mostly decomposed and could no longer be recovered. Controlled incubation at 4 °C significantly preserved ascospore viability, maintaining a 25.2% germination rate after eight weeks and retaining 42.4% of its initial germination capacity. In contrast, incubation at 23 °C led to a drastic reduction in viability, with germination dropping to just 0.7%—a 98.7% decline. These findings suggest that Florida’s warm off-season climate, combined with widespread tillage practices, may naturally limit the survival of *P. maydis* inoculum between growing seasons. Conversely, our results indicate that cold conditions, such as refrigeration or cooler seasonal temperatures, can help preserve ascospore viability following leaf detachment. These findings clarify the duration of *P. maydis* viability following leaf detachment in Florida conditions and provide a foundation for developing region-specific management strategies to disrupt the pathogen’s life cycle between growing seasons. Future research should focus on evaluating the role of microbial activity in pathogen survival and identifying potential alternative hosts that may facilitate pathogen survival during the off-season. Gaining a deeper understanding of the interactions among tillage practices, regional climate, and the biology of *P. maydis* will be critical for developing sustainable, region-specific management strategies to mitigate the impact of tar spot in Florida’s maize production systems, particularly if the disease becomes epidemic in the future.

## Figures and Tables

**Table 1:** Statistical comparison of germination probabilities between the initial (week two) and final (week eight) time points for each treatment.

Treatment	Initial Germination (%)	Germination Decline (%)	Odds Ratio (%) *^a^*	Odds Ratio SE *^b^*	*p*-Values ^*c*^
Muck	10.9	49.9	50.1	30.1	0.2501
Rocky	13.4	88.9	11.1	11.9	0.0403
4 °C	44.2	97.3	42.4	14.4	0.0113
23 °C	20.6	57.3	2.7	2.0	<0.0001

*^a^* Odds ratio represents the proportional change in germination from the initial to the final time point. *^b^* SE indicates standard errors. *^c^* *p*-values indicate the significance of changes in germination probabilities between the two time points.

## Data Availability

The original contributions presented in this study are included in the article. Further inquiries can be directed to the corresponding author.
